# Identification of FOXP1 Deletions in Three Unrelated Patients with Mental Retardation and Significant Speech and Language Deficits

**DOI:** 10.1002/humu.21362

**Published:** 2010-11

**Authors:** Denise Horn, Johannes Kapeller, Núria Rivera-Brugués, Ute Moog, Bettina Lorenz-Depiereux, Sebastian Eck, Maja Hempel, Janine Wagenstaller, Alex Gawthrope, Anthony P Monaco, Michael Bonin, Olaf Riess, Eva Wohlleber, Thomas Illig, Connie R Bezzina, Andre Franke, Stephanie Spranger, Pablo Villavicencio-Lorini, Wenke Seifert, Jochen Rosenfeld, Eva Klopocki, Gudrun A Rappold, Tim M Strom

**Affiliations:** 1 Institute of Medical Genetics, Charité, University Medicine of BerlinBerlin, Germany; 2 Department of Molecular Human Genetics, Ruprecht-Karls-UniversityHeidelberg, Germany; 3 Institute of Human Genetics, Helmholtz Zentrum München, German Research Center for Environmental HealthNeuherberg, Germany; 4 Institute of Human Genetics, Ruprecht-Karls-UniversityHeidelberg, Germany; 5 Institute of Human Genetics, Technische Universität MünchenMunich, Germany; 6 Wellcome Trust Centre for Human Genetics, University of OxfordOxford, UK; 7 Department of Medical Genetics, Institute of Human Genetics, University of TübingenTübingen, Germany; 8 Institute of Human Genetics, Rheinische Friedrich-Wilhelms-UniversityBonn, Germany; 9 Institute of Epidemiology, Helmholtz Zentrum München, German Research Center for Environmental HealthNeuherberg, Germany; 10 Heart Failure Research Center, Department of Experimental Cardiology, Academic Medical Center, University of AmsterdamAmsterdam, The Netherlands; 11 Institute for Clinical Molecular Biology, Christian-Albrechts-University zu KielKiel, Germany; 12 Praxis für HumangenetikBremen, Germany; 13 Cologne Center for Genomics, Universität zu KölnCologne, Germany; 14 Faculty of Biology, Chemistry, and Pharmacy, Free University of BerlinBerlin, Germany; 15 Department of Audiology and Phoniatrics, Charité, University Medicine of BerlinBerlin, Germany

**Keywords:** FOXP1, mental retardation, copy number variations, language and speech deficits

## Abstract

Mental retardation affects 2-3% of the population and shows a high heritability. Neurodevelopmental disorders that include pronounced impairment in language and speech skills occur less frequently. For most cases, the molecular basis of mental retardation with or without speech and language disorder is unknown due to the heterogeneity of underlying genetic factors. We have used molecular karyotyping on 1523 patients with mental retardation to detect copy number variations (CNVs) including deletions or duplications. These studies revealed three heterozygous overlapping deletions solely affecting the forkhead box P1 (FOXP1) gene. All three patients had moderate mental retardation and significant language and speech deficits. Since our results are consistent with a de novo occurrence of these deletions, we considered them as causal although we detected a single large deletion including FOXP1 and additional genes in 4104 ancestrally matched controls. These findings are of interest with regard to the structural and functional relationship between FOXP1 and FOXP2. Mutations in FOXP2 have been previously related to monogenic cases of developmental verbal dyspraxia. Both FOXP1 and FOXP2 are expressed in songbird and human brain regions that are important for the developmental processes that culminate in speech and language. ©2010 Wiley-Liss, Inc.

## INTRODUCTION

Mental retardation (MR) is defined as a significant impairment of cognitive and adaptive functions with an onset before the age of 18 years (Ropers, 2008). Based on the assessment of patients’ intelligence quotients (IQs), MR is usually classified into mild (IQ 50-70), moderate (IQ > 35) and severe (IQ > 20) forms. It has been shown that the causes of the disorder include environmental and genetic factors (Inlow and Restifo, 2004), still for most cases, the pathological basis remains unexplained. The high degree of heritability of MR is highlighted by the estimation that up to 50% of severe cases are caused by genetic abnormalities (Inlow and Restifo, 2004). Due to the heterogeneity of the underlying genetic factors, the identification of MR candidate genes still remains difficult to date. A promising approach to detect small chromosomal copy number variants (CNVs) in the genome is the use of molecular karyotyping techniques (Marshall, et al., 2008). CNVs have been shown as the underlying cause or susceptibility factors for a variety of autism spectrum disorders and conditions associated with MR (Berkel, et al., 2010; Marshall, et al., 2008; Sebat, et al., 2007; Zweier, et al., 2010). Array-based comparative genomic hybridization has also enabled the detection of interstitial submicroscopic copy number alterations in about 10 % of patients with MR (de Vries, et al., 2005). Here we present the identification of overlapping heterozygous deletions affecting the *FOXP1* (MIM# 605515) gene in three unrelated patients with MR and significant speech and language disorder.

## MATERIALS AND METHODS

### Oligonucleotide arrays

CNV data were generated in different institutions within the MRNET consortium (Supp. Table S1). Patient 1 was part of a cohort of 387 patients investigated with Infinium Human550-Quad and Human610-Quad arrays (Illumina). Intensity data were normalized as described previously (Wagenstaller, et al., 2007). Segmentation was performed with circular binary segmentation as implemented in the R-package ‘DNAcopy'. Patient 2 was part of a cohort of 188 patients investigated using whole genome oligonucleotide 244K arrays (Agilent Technologies, Santa Clara, CA). Image data were analyzed using Feature Extraction 9.5.3.1 and CGH Analytics 3.4.40 software (Agilent Technologies, Santa Clara, CA) with the following analysis settings: aberration algorithm ADM-2; threshold: 6.0; window size: 0.2 Mb; filter: 5probes, log2ratio = 0.29. Patient 3 belonged to a cohort of 184 patients analyzed with genome-wide human SNP 6.0 arrays (Affymetrix, Santa Clara, CA). Analysis of data was performed using the Genotyping Console Software 3.0 (Affymetrix). For the detection of genomic deletions and duplications, automated analysis by Segment Reporting Tools was used. Regions showing at least 5-10 aberrant neighboring SNPs / markers and having a size of at least 100 kb were classified as being significant. Additional CNV data of 764 MR patients from other institutions not listed here were obtained through the database of the MRNET (www.german-mrnet.de).

### Control populations

Controls consisted of 1146 individuals from popgen, 813 individuals of a population-based cohort (KORA study), 972 patients with cardiac ischemia (AGNES study), 482 patients with early-onset lung cancer (LUCY study), and 691 long-lived individuals (LLI study).

### Breakpoint identification

PCR reactions on genomic DNA level were performed to amplify the junction fragments that spanning the telomeric and centromeric breakpoints. Fragments were directly sequenced with the respective forward and reverse primer: B35_FOXP1F/B8_FOXP1R (patient 1): 5′-atgctgaaggtggaatggg-3′, 5′-ggccacatacgtgttgtcag-3′; O06_for/Z02_rev (patient 2): 5′-cgttgccagctcaaggttat-3′, 5′-taagtgtgtgcgaagccaag-3′; bp_FOXP1_3for/rev (patient 3): 5′-gcacctgaccctctagctca-3′, 5′-ggttcagccactggtctttc-3′.

### Fluorescence *in situ* hybridization (FISH)

Preparation of chromosome metaphases of patient 3 and his parents and FISH were performed according to standard protocols using BAC DNA probes RP11-215K24, RP11-154H23, CTD-3121O8 (Invitrogen, Darmstadt, Germany) and RP11-788D09 (BACPAC Resource Center, Oakland, USA).

### Mutation screening

FOXP1 (NM_032682.4) exons were PCR amplified using intronic primers and investigated with 2 different methods: 197 DNAs were investigated by direct sequencing, 772 DNAs were analyzed using dye-binding/high-resolution DNA melting point analysis (LightScanner HR I 384, Idaho Technology). Genotyping of the controls were performed on a MALDI-TOF mass-spectrometer (Sequenom MassArray system) using the homogeneous mass-extension (hME) process for producing primer extension products. Primers were designed with ExonPrimer (http://ihg.helmholtz-muenchen.de/ihg/ExonPrimer.html) and are available on request. Nucleotide numbering reflects cDNA numbering with +1 corresponding to the A of the ATG translation initiation codon.

### SLIC study

The SLIC probands were selected from samples ascertained by the Newcomen Centre at Guys Hospital and by the Manchester Language Study (The SLI Consortium, 2002) and all had severe language impairments with language skills more than 1.5 SD below the normative mean for their chronological age in combination with full IQ scores at least 0.7 SD below that expected for their age. IQ scores are derived from the Wechsler Intelligence Scales for Children (WISC-III). The 46 (16 males, 30 females; average age: 11 years and 3 months) individuals would not meet strict SLI diagnostic criteria as individuals with IQ problems are usually excluded. Nonetheless all probands had severe language impairments which require special schooling arrangements and continued support.

## RESULTS

### *FOXP1* deletions

In a collaborative effort we performed a genome-wide microarray scan for CNVs in a German cohort of 1523 unrelated patients with unexplained mental retardation. Standard diagnostic tests like chromosomal karyotyping, fragile X testing and subtelomeric screenings were performed in most cases to rule out known causes of MR. The recruitment of patients was part of the German Mental Retardation Network (MRNET) study. Approval for the study was obtained by the ethical review boards of the participating institutions and informed written consent was obtained from all participants.

**Figure 1:**
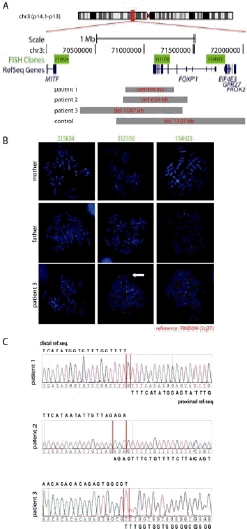
*FOXP1* deletions in patients with MR. (A) Schematic presentation of the position and size of the deleted regions (grey bars) at chromosome 3p14.1 in three patients with MR and in a control individual. Only the *FOXP1* gene (NM_032682, minus strand) is affected. The coding exons are indicated by larger vertical lines compared to the non-coding exons. (B) Results of fluorescence *in situ* hybridization (FISH) shown for patient 3 and his parents. The positions of the BAC clones RP11-215K24 and RP11-154H23 flanking the deletion, as well as CTD-3121O8 located within the deletion (green signals) are given in Figure 1A. BAC clone RP11-778D09 was used as a reference probe located on chromosome 3q27 (red signals). The white arrow indicates the missing signal for clone CTD-3121O8 on one of the patient's chromosomes 3 while the signal is present in both chromosomes of the parents. (C) Breakpoint characterization by sequencing of junction fragments amplified by PCR on genomic DNA of the patients. In patient 1, the distal breakpoint was located at position 70,807,767, the proximal breakpoint at position 71,305,965 with a deletion size of 498,198 bp. In patient 2, the two breakpoints were located within a microhomology of three nucleotides (AGA) at the telomeric side at position 70,778,067-70,778,070 and the centromeric side at 71,437,354-71,437,357 resulting in a deletion size of 659,287 bp. For patient 3, the telomeric breakpoint is located at position 70,341,246-70,341,247 and the centromeric breakpoint at position 71,388,173-71,388,174 with a deletion size of 1,046,927 bp. All positions are given according to hg18, UCSC Human Genome March 2006.

Copy number analysis revealed overlapping deletions at chromosome 3p14.1 affecting solely the *FOXP1* gene in three unrelated cases, two males and one female aged between 5.5 to 7 years. Deletion sizes of 498 kb, 659 kb and 1047 kb included all but the first of the coding exons in patient 1 (Wagenstaller, et al., 2007) and the entire coding region in the other two patients 2 and 3 (Figure 1A). The deletions were verified by fluorescence *in situ* hybridization (FISH; Figure 1B) or quantitative real-time PCR (data not shown). DNA analysis of the unaffected parents by molecular karyotyping and FISH indicated that patients 2 and 3 carried *de novo* deletions, while it could only be shown that the mother of patient 1 does not carry the deletion since the father was not available for investigation. We found that the deleted alleles were of paternal origin in all three patients. Sequencing of the coding exons of the remaining *FOXP1* allele did not reveal any sequence variation compared to the annotated reference sequence (NM_032682). Characterization of the breakpoints was achieved by amplification and sequencing of the respective junction fragments (GenBank EF504249, HM124444, and GU980955; Figure 1C).

For the deletion in patient 1, the distal breakpoint is located within a long interspersed nuclear element (LINE) repeat (L1/L1PA5) while the proximal breakpoint lies in a MER1-type repeat (MER58A). No repetitive sequences are present at the breakpoint loci in patient 2, however, microhomoloy of 3 bases (AGA) is observed at the breakpoints. The distal breakpoint of the deletion in patient 3 is located in a short interspersed nuclear element (SINE) while repetitive sequences are missing at the proximal breakpoint. The absence of homologous regions at the breakpoints suggests that the deletions were likely generated by double-strand breaks and non-homologous end-joining.

### Control populations

To assess the significance of these findings, we checked the *FOXP1* region for CNVs in the *Database of Genomic Variants* (DGV) (Iafrate, et al., 2004) and in 4104 ancestrally matched controls which have been investigated with different types of high-density SNP arrays containing at least 500,000 SNPs (Supp. Table S1). No CNVs affecting the coding region were found in the DGV database. Copy number analysis in the 4104 control individuals revealed the presence of a single large 3p14.1p13 deletion of approximately 1.3 Mb affecting *FOXP1, EIF4E3* (MIM# 609896), *PROK2* (MIM# 607002) and *GPR27* (MIM# 605187) in an individual of the LLI study, with no indications of a comparable MR phenotype. Detailed clinical data on intellectual abilities were lacking and further investigations not possible due to the design of the study. In particular, we could not investigate the presence of somatic mosaicism. Application of Fisher's exact test on these data, not considering the controls contained in the DGV database, resulted in a nominal p-value of 0.06. Thus, although the absence of deletions in the unaffected parents is consistent with a role of *FOXP1* in the clinical findings of the patients incomplete penetrance of deletions in this chromosomal interval cannot be ruled out.

### Speech and language development

**Figure 2:**
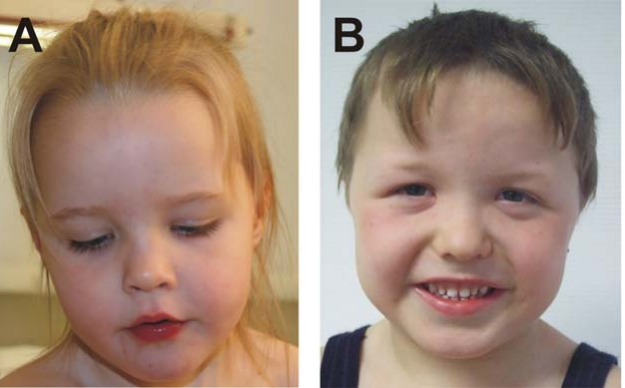
Facial Phenotype of patients 2 and 3. Consistent facial features of patients showing broad and prominent forehead, frontal hair upsweep, and a short nose. (A) Patient 2, at the age of 4 years and 3 months. (B) Patient 3, at the age of 6 years, had in addition sparse lateral eye brows and down-slanting palpebral fissures.

Detailed clinical investigation of the three patients revealed that they present moderate MR in combination with a general developmental delay (Table 1). In all patients, the non-verbal IQ score was assessed as < 50 (3 SD below the mean). Speech and language development was estimated in the same range. All three patients started to speak at age of 3.5 years and used only combinations of two words at ages 5, 5.5 and 7 years, respectively. In all patients, the productive and receptive vocabulary came up to less than 100 words. Expressive language was more affected than receptive abilities. Dysgrammatism and very poor speech articulation with difficulties producing consonants at the beginning of words was present in all patients. Two showed oromotoric problems including difficulties with lip protrusion. In infancy, patients had a tendency to keep their mouth open and patient 2 suffered from swallowing difficulties. In addition, all showed considerably retarded gross-motor development with unsupported walking as late as 24 to 36 months. Brain magnetic resonance imaging and electroencephalography did not reveal any abnormalities. None of the patients showed sensorineural hearing loss. Ophthalmologic testing disclosed moderate myopia only in patient 1. Upon physical examinations, normal growth parameters regarding height and occipitofrontal head circumference were documented for two patients but presence of obesity was striking in both. Consistent craniofacial anomalies seen in patients 2 and 3 included broad and prominent forehead and frontal hair upsweep (Figure 2).

**Table 1:** Clinical description of *FOXP1* deletion patients

	Patient 1	Patient 2	Patient 3
**General features**			
Age of last assessment	7 years	5.5 years	6 years
Sex	Male	Female	Male
Occipitofrontal head circumference (SD)	+1.2	+0.8	+1.3
Height (SD)	+1.4	+0.6	-0.4
Weight (SD)	+0.5	+2.7	+2.5
Facial gestalt	no dysmorphisms	Prominent forehead, frontal hair upsweep	Prominent forehead, frontal hair upsweep
Non-verbal performance IQ	<50	<50	50
Gross motor delay	+	+	+
Age at sitting	n.d.	12 months	8 months
Age at walking	24 months	36 months	24 months
Swallowing difficulties	n.d.	+	-
Oromotor problems (e.g. lip protrusion, tongue elevation)	+	+	-

**Speech and language development**			
First vocalizing at age of	n.d.	4 months	12 months
First words at age of	3.5 years	3.5 years	3.5 years
Combined words at age of	7 years	5 years	5.5 years
Articulation problems	+	+	+
Poor grammar	+	+	+

n.d., not documented

### *FOXP1* point mutations

In order to detect possible *FOXP1* point mutations, we sequenced the coding exons in 883 patients with MR from the MRNET database and discovered eight different non-synonymous, three synonymous and nine non-coding variants (Table 2). All variants were genotyped in up to 676 unrelated healthy controls. Three of the identified coding variants, p.Ser5Pro, p.Pro215Ala and p.Thr390Ser were also present at similar frequencies in healthy matched controls, but none of the coding variants was found in the HapMap or the NCBI dbSNP database. Five of the non-synonymous variants were transmitted through an apparently unaffected parent; for the other variants, heredity could not be established as either one or both parents were not available (Table 2). Variants transmitted by an unaffected parent are usually classified as non-pathogenic. Still, presuming a multigenic threshold model for MR, disease relevance cannot be completely ruled out. To uncover the neurobiological significance of these variants, investigation of the functional effects of the putative mutations is needed.

**Table 2:** Summary of *FOXP1* variants identified in controls and in patients with mental retardation

Variants		MR patients					Controls		
			Genotypes			Status of inheritance	Genotypes		
			11	12	22		11	12	22
**Non-synonymous variants**									
c.13T>C	p.Ser5Pro	Exon 6	882	1	0	maternal	674	2	0
c.226_228dupCAG	p.Gln76dup	Exon 7	882	1	0	maternal	676	0	0
c.301A>G	p.Met101Val	Exon 8	882	1	0	n.a.	667	0	0
c.643C>G†	p.Pro215Ala	Exon 10	882	1	0	maternal	336	2	0
c.781T>C	p.Ser261Pro	Exon 11	882	1	0	n.a.	676	0	0
c.1168A>T	p.Thr390Ser	Exon 15	882	1	0	maternal	675	1	0
c.1709A>G	p.Asn570Ser	Exon 19	882	1	0	paternal	338	0	0
c.1790A>C	p.Asn597Thr	Exon 20	882	1	0	n.a.	676	0	0

**Synonymous variants**									
c.768G>A	p.Thr256Thr	Exon 11	882	1	0	n.a.	673	0	0
c.1188G>A	p.Ser396Ser	Exon 15	882	1	0	maternal	674	0	0
c.1515C>T	p.Asn505Asn	Exon 17	882	1	0	maternal	674	0	0

**Non-coding variants**									
c.1-5G>A	5′UTR	882	1	0	paternal	674	0	0	
c.180+49T>C	Intron 6	80	30	1	not tested (dbSNP rs2037474)				
c.181-29G>A†	Intron 6	875	7	1	1 x mat, 2 x pat, 1 x mat + pat, 4 x n.a.	653	21	0	
c.181-30C>T	Intron 6	882	1	0	n.a.	676	0	0	
c.664+11A>G	Intron 10	882	1	0	maternal	674	0	0	
c.664+6C>T	Intron 10	882	1	0	n.a.	674	0	0	
c.975-14A>G	Intron 12	882	1	0	maternal	673	1	0	
c.1889+20A>C†	Intron 20	95	15	1	not tested (dbSNP rs7638391)				
c.1890-15G>T†	Intron 20	110	1	0	not tested (dbSNP rs7639736)				

Listed is the number of individuals carrying the respective homozygous or heterozygous genotypes (11, 12 or 22; GenBank NM_032682.4). Patients and matched healthy controls were of German origin.

† also found by Vernes *et al*, 2009.

MR, mental retardation; n.a., parents not available; mat, maternal; pat, paternal. Nucleotide numbering reflects cDNA numbering with +1 corresponding to the A of the ATG translation initiation codon.

Since the core phenotype of the three patients carrying *FOXP1* deletions consisted of MR with significant speech and language deficits, we additionally sequenced *FOXP1* coding exons in DNA of 40 patients with a tentative diagnosis of Angelman syndrome (without microcephaly and negative for *SNRPN* imprinting). We also sequenced the DNA of 46 probands from the Specific Language Impairment Consortium (SLIC) collection (The SLI Consortium, 2002). For both groups, no sequence variants in the coding region *of FOXP1* were detected.

## DISCUSSION

This is the first report of 3p14.1 deletions that solely affect the *FOXP1* gene in three unrelated patients with moderate mental retardation in combination with a significant impairment of speech and language abilities. We investigated a large control population and the apparent finding of a large 3p14.1p13 deletion in this cohort affecting *FOXP1* and further genes illustrates the difficulties to assess the significance of rare mutational events in extremely heterogeneous diseases and may point to incomplete penetrance. The additional findings of obesity and mild craniofacial anomalies (prominent forehead and frontal hair upsweep) in two patients might be part of the clinical spectrum associated with *FOXP1* deletions. A *de novo* deletion of this chromosomal interval on 3p14. 1p13 affecting part of *FOXP1* and three additional genes (*EIF4E3, PROK2* and *GPR27*) has been previously described in a boy with multiple abnormalities including speech and developmental delay (Pariani, et al., 2009). Compared to the clinical findings in our patients, there is a clear overlap with regard to the speech and developmental delay suggesting that haploinsufficiency of FOXP1 is indeed causative for this phenotype. As our patients did not show any of the other clinical signs like contractures, hypertonia and blepharophimosis, present in the boy carrying the larger 3p14.1p13 deletion (Pariani, et al., 2009), these might rather be caused by the haploinsufficiency of the additionally affected genes.

FOXP1 belongs to a functionally diverse family of forkhead box (FOX) transcription factors that are all characterized by a highly conserved FOX domain. *FOX* genes have been shown to play important roles in diverse cellular functions including metabolic and developmental processes (Carlsson and Mahlapuu, 2002). While the function of FOXP1 during blood cell, lung and heart formation has already been addressed (Hu, et al., 2006; Shi, et al., 2004; Shu, et al., 2001; Wang, et al., 2004), the role of FOXP1 in neuronal processes, in particular brain development, is still unclear. In a mouse mutant model, Foxp1 has recently been shown to be an important accessory factor in Hox transcriptional output, thus regulating motor neuron diversification and connectivity to target muscles (Dasen, et al., 2008; Rousso, et al., 2008). These findings are of particular interest with regard to the gross-motor and oromotor deficits seen in our patients with *FOXP1* deletions.

Currently, four *FOX* genes (*FOXC1* [MIM# 601090], *FOXC2* [MIM# 602402], *FOXP2* [MIM# 605317] and *FOXP3* [MIM# 300292]) are listed in the Online Mendelian Inheritance in Man (OMIM) database that have been shown to be causative for human diseases (Hannenhalli and Kaestner, 2009). With regard to structure and expression profiles, the most interesting gene related to *FOXP1* is *FOXP2*. Rare mutations of *FOXP2* have been described in individuals having expressive and receptive language and speech deficits generally described as developmental verbal dyspraxia (Hurst, et al., 1990; Lai, et al., 2001; MacDermot, et al., 2005; Marshall, et al., 2008). It is striking, that patients with *FOXP1* deletions show a comparable speech and language deficit. Most of the mutations affecting *FOXP2* are deletions and maternal uniparental disomies. In almost all cases, the affected allele is of paternal origin suggesting differential parent-of-origin expression of *FOXP2* in human speech development (Marshall, et al., 2008). Of note, there is some parallelism as the three *FOXP1* deletions we describe are also of paternal origin.

A functional relationship between Foxp1 and Foxp2 has been previously demonstrated in mouse, as they form homo- and heteromers necessary for efficient DNA binding (Marshall, et al., 2008). *FoxP1* and *FoxP2* also show overlapping expression patterns within brains of zebra finches and fetal human brains, particularly in subcortical regions that play important roles in sensorimotor integration and coordinated movements important for vocalization and speech respectively (Teramitsu, et al., 2004). From these findings it was speculated that FOXP1 might play an important role in the development of brain circuits that coordinate fine sequential motor control required for articulation.

Although the mean non-verbal IQ of the members of the large family carrying *FOXP2* mutation was lower than of the unaffected members, non-verbal intellectual impairment could not be considered characteristic of the phenotype associated with this *FOXP2* mutation (Lai, et al., 2001). This is in contrast to the clinical findings in our patients with *FOXP1* deletions who, in addition to their speech and language disabilities, exhibit significantly reduced non-verbal IQ scores. This may indicate that additional distinct functions of FOXP1 exist, presumably acting on a more global level in neuronal development compared to FOXP2. This would be in line with results from a previous study on 49 patients with developmental verbal dyspraxia and normal intelligence, where no causative *FOXP1* mutations could be detected (Vernes, et al., 2009).

In conclusion, we report a previously unknown cause of MR associated with significant speech and language disorder defined by *FOXP1* deletions. We propose that haploinsufficiency of FOXP1 leads to abnormal development of neural structures that coordinate general cognitive and psychomotoric as well as verbal abilities.

## Figures and Tables

**Supp. Table S1:** Setup of molecular karyotyping analyses in patients with MR and control individuals

Cohort	Arrays	Data analysis; thresholds / settings	Individuals

Patients MRNET			
**Munich**	Infinium Human550-Quad and Human610-Quad (Illumina)	Data were normalized according to Wagenstaller et al. (2007).	
		Segmentation was performed with circular binary segmentation as implemented in the R-package ‘DNAcopy’.	387
**Berlin**	Whole-genome oligonucleotide 244K array (Agilent Technologies, Santa Clara, CA)	Feature Extraction 9.5.3.1 and CGH Analytics 3.4.40; aberration algorithm ADM-2, threshold: 6.0; window size: 0.2 Mb; filter: 5 probes; log2ratio = 0.29	188
**Heidelberg**	Genome-wide human SNP Array 6.0 (Affymetrix, Santa Clara, CA)	Segment Reporting Tools of the Genotyping Console 3.0 software; only CNVs > 100 kb with at least 5-10 aberrant SNPs	184
Other*			764
**Sum**			**1523**

**Control individuals**			

**popgen study**	Genome-wide human SNP Array 6.0 (Affymetrix, Santa Clara, CA)	CNVineta (http://www.ikmb.uni-kiel.de/cnvineta)	1146
**KORA study**	Infinium Human550-Quad (Illumina)	Data were normalized according to Wagenstaller et al. (2007).	813
**AGNES study**	Infinium Human6100-Quad (Illumina)	Segmentation was performed with circular binary segmentation as implemented in the R-package ‘DNAcopy’.	972
**LUCY study**	Infinium Human550-Quad (Illumina)		482
**LLI study**	Genome-wide human SNP Array 6.0 (Affymetrix, Santa Clara, CA)	CNVineta (http://www.ikmb.uni-kiel.de/cnvineta)	691
**Sum**			**4104**

Given are the setups of the molecular karyotyping performed in MR patients by the authors' institutions.

* Additional CNV data of MR patients from other institutions (not listed here) were obtained through the database of the MRNET (http://www.german-mrnet.de); popgen study (http://www.popgen.de); KORA study (http://www.helmholtz-muenchen.de/kora); MR, mental retardation.
